# Protocol for a drugs exposure pregnancy registry for implementation in resource-limited settings

**DOI:** 10.1186/1471-2393-12-89

**Published:** 2012-09-03

**Authors:** Ushma Mehta, Christine Clerk, Elizabeth Allen, Mackensie Yore, Esperança Sevene, Jan Singlovic, Max Petzold, Viviana Mangiaterra, Elizabeth Elefant, Frank M Sullivan, Lewis B Holmes, Melba Gomes

**Affiliations:** 1 Independent Pharmacovigilance Consultant, Cape Town, Kenilworth, 7708, South Africa; 2 Department of Epidemiology and Disease Control, School of Public Health, University of Ghana, Legon, Ghana, South Africa; 3 Division of Clinical Pharmacology, Department of Medicine, University of Cape Town, Cape Town, South Africa; 4 Stanford University School of Medicine, 291 Campus Drive, Stanford, CA, 94305, USA; 5 Department of Pharmacology, Eduardo Mondlane University, Manhiça Foudation, Maputo, Mozambique; 6 Data Management Consultant, Libochovany, 41103, Czech Republic; 7 Centre for Applies Biostatistics, Department of Medicine, Sahlgrenska Academy, University of Gothenburg, Gothenburg, Sweden; 8 World Health Organization, 1211 Avenue Appia, Geneva, 27, Switzerland; 9 Centre de Référence sur les Agents Tératogènes (CRAT), Groupe Hospitalier Universitaire Est, Hôpital Armand Trousseau, 26, av du Dr Netter, 75571, Paris cedex, 12, France; 10 Harrington House, 8 Harrington Road, Brighton, BN1 6RE, UK; 11 Genetics Unit, Massachusetts General Hospital for Children, 175 Cambridge Street, 504, Boston, MA, 02114, USA

**Keywords:** Pregnancy Registry, Congenital anomaly, Pharmacovigilance, Teratogenicity, Drug exposure, Antiretrovirals, Antimalarials, Birth defects, Neonates, Safety, Resource-limited settings

## Abstract

**Background:**

The absence of robust evidence of safety of medicines in pregnancy, particularly those for major diseases provided by public health programmes in developing countries, has resulted in cautious recommendations on their use. We describe a protocol for a Pregnancy Registry adapted to resource-limited settings aimed at providing evidence on the safety of medicines in pregnancy.

**Methods/Design:**

Sentinel health facilities are chosen where women come for prenatal care and are likely to come for delivery. Staff capacity is improved to provide better care during the pregnancy, to identify visible birth defects at delivery and refer infants with major anomalies for surgical or clinical evaluation and treatment. Consenting women are enrolled at their first antenatal visit and careful medical, obstetric and drug-exposure histories taken; medical record linkage is encouraged. Enrolled women are followed up prospectively and their histories are updated at each subsequent visit. The enrolled woman is encouraged to deliver at the facility, where she and her baby can be assessed.

**Discussion:**

In addition to data pooling into a common WHO database, the WHO Pregnancy Registry has three important features: First is the inclusion of pregnant women coming for antenatal care, enabling comparison of birth outcomes of women who have been exposed to a medicine with those who have not. Second is its applicability to resource-poor settings regardless of drug or disease. Third is improvement of reproductive health care during pregnancies and at delivery. Facility delivery enables better health outcomes, timely evaluation and management of the newborn, and the collection of reliable clinical data. The Registry aims to improves maternal and neonatal care and also provide much needed information on the safety of medicines in pregnancy.

## Background

Efforts to increase access to medicines for major diseases such as malaria, HIV/AIDS, pneumonia and tuberculosis, have accelerated. Novel life-saving therapies such as artemisinin-based combination therapies (ACTs), antiretrovirals (ARVs), anti-infectives and vaccines have been introduced on a large scale [1]. However, the safety of some of these therapies during pregnancy is unknown.

In some infections (HIV/AIDS, malaria), failure to treat effectively may result in death, disease transmission or poor outcomes for the mother and/or the baby. However the medicines themselves may cause toxicity, including birth defects. Therefore, knowledge about the risks of medicines during each trimester of pregnancy is essential in order to assist patient management.

Animal studies suggest that the artemisinins as a class (artesunate, dihydroartemisinin, artemether and arteether) interfere with red blood cell formation and can cause fetal loss at low dose levels. If administered during early embryogenesis, congenital malformations may also be induced [2,3]. The time window of sensitivity observed in animal studies would correspond in humans to part of the first trimester, during organogenesis. However, the period of erythroblast expansion in rodents that is sensitive to the artemisinin damage occurs over a very short period of about one day. In primates and humans it occurs over a more prolonged period, and the usual short three-day course of artemesinin treatment may not be long enough to cause extensive damage. This may be the reason why no such cases have been reported in humans [4,5].

With efavirenz (EFV), central nervous system malformations have been observed in primates at doses comparable to systemic human exposure and although retrospective case reports in humans exposed to EFV show similar defects, a causal relationship has not been established. A recent meta-analysis of data describes the incidence of neural tube defects in the babies of women receiving EFV during the first trimester (0.7%) to be no higher than the incidence in babies born to women on ARV regimens not containing EFV [6]. Although reassuring, the limited sample of observations does not eliminate this risk. Concerns have also been raised about the effect of tenofovir on growth restriction and severe bone toxicity in pre-clinical primate and clinical studies [7].

The WHO treatment guidelines for malaria and HIV/AIDS reflect these concerns about potential risk during pregnancy [8,9]. In practice, pregnant women in countries with high burden of infections are likely to be exposed to both medicines since the number of pregnancies among HIV-positive women already on ARVs is increasing in both developed [10] and developing countries [11] and many pregnancies are unplanned. At the same time, changes in drugs delivered across the care continuum (antenatal, delivery, postpartum) pose operational challenges in high-burden low-resource settings. WHO’s 2012 programmatic update for prevention of mother to child transmission (PMTCT) points out that, although concerns remain about EFV and monitoring is necessary, triple ARV treatment under Option B and Option B + (CD4 testing needed and desirable, respectively) has benefits at the primary care level. It provides greater assurance that women in need of treatment receive a fully suppressive regimen that minimizes risk of infant infection and maximizes their own health benefit [12]. In confirmed and unconfirmed diagnoses of malaria, the artemisinins are being used extensively without knowledge of pregnancy status in women of childbearing age. Thus the likelihood of first trimester exposures to medications for malaria and HIV has increased and is expected to increase even more [13], making a pregnancy exposure registry essential.

Guidelines distinguish pregnancy registries from other post-marketing surveillance techniques in that pregnant women are enrolled before the outcome of the pregnancy is known[14], and many outcomes can be monitored. If the purpose of the registry is to establish any additional risk of birth defects for a particular drug, pregnancy outcomes of women exposed to the drug/s must be compared with outcomes of women who do not have the disease and were not exposed to the medications of interest. The possibility of confounding by indication might also be addressed by assessing differences in risk associated with different medicines used for the same clinical condition, e.g. different ARVs for HIV. Moreover, the data would allow for the assessment of risk of multiple infections in pregnancy including sexually transmitted diseases, tuberculosis, malaria and HIV/AIDS. Prospective monitoring is critical to reduce the likelihood of selection and recall bias and to obtain reliable information on drug exposure and other risk factors. In most disease-endemic settings where ARVs and ACTs are used, the background risk of congenital anomalies and other adverse pregnancy outcomes is not known. [15]

We describe a Pregnancy Registry protocol to provide evidence on the risk of increased prevalence of clinically important malformations at birth associated with medicines to which women might be exposed. The protocol is neither disease nor drug-specific; this confers substantial advantages for wider use of the same dataset. Its methods, case record forms (CRFs) and training materials (including a DVD showing how to conduct a surface examination of a newborn) have been tested for feasibility in five countries (four in Africa and one in South America). They have been refined as a consequence. The approach is integrated within the reproductive health system of the country, specifically ANC clinics and labor/delivery facilities. The protocol builds on the fact that in most African countries 90% of women access health care during pregnancy, and malaria treatment, HIV counseling and testing and PMTCT programmes have good links with reproductive health services. It assumes that individual countries and sentinel sites will contribute to a pooled WHO database on safety of medications in pregnancy, and that materials developed by WHO for use are available to any country wishing to join, on condition that there is a commitment to train the staff to use these materials to obtain reliable data on drug exposure and to conduct a systematic surface examination of the newborn. The Registry builds capacity within the health system to improve maternal and neonatal care as well as to serve as a sentinel surveillance system for the safety of medicines used in pregnant women.

### The specific objectives of the WHO Pregnancy Registry are

1. To build capacity to obtain reliable information on obstetric, medical, and drug history during pregnancy and diagnose, assess, monitor and manage pregnancy and the outcomes of pregnancy including congenital malformations, stillbirths and prematurity.

2. To quantify the baseline risk of major congenital malformations in the absence of drug exposure during the course of pregnancy.

3. To quantify the risk of major congenital malformations associated with exposure to medicines during the course of pregnancy.

4. To identify other obstetric, therapeutic and clinical factors that may contribute to the risk of major congenital anomalies and other adverse birth outcomes in pregnant women.

5. To support a culture of drug safety awareness among women and their providers in participating countries and avoid preventable adverse drug-related pregnancy outcomes.

6. To develop an ongoing surveillance system of maternal and newborn health that strengthens the health system to improve maternal and neonatal outcomes.

The primary endpoint is major external/visible congential anomalies and the secondary endpoint is other adverse birth outcomes including stillbirth, prematurity and neonatal death within 24 hours of birth.

## Methods/Design

This Pregnancy Registry is a prospective observational cohort study that enrols pregnant women at their first antenatal visit to a selected health facility. To obtain a more accurate medical and drug exposure history, early enrollment is desirable.

### Study Population

The population of interest is pregnant women at selected antenatal clinics (ANCs). These clinics are chosen because of their location in areas with a high prevalence of the disease of interest (i.e. where women are likely to be exposed to the drug/s in question). In addition, women presenting to these facilities should be very likely to initiate antenatal care early in pregnancy and deliver at the facility. All pregnant women seeking care for the first time during their current pregnancy are eligible. However, if there is a very high case-load at a facility, a random sampling method might be devised. Thus, the number of women recruited at any facility will depend on practical considerations such as available staff capacity, attendance rates, facility delivery rates, and willingness of women to consent to being followed to term.

Country-sites fulfilling the following criteria would be ideally placed to contribute to the Registry database.

• Strong commitment to the Registry from the relevant departments of the Ministry of Health, particularly Reproductive health and disease control programmes (eg malaria and HIV/AIDS).

• Written agreement to pool all data derived from the Pregnancy Registry into the WHO International Registry database.

• A moderate to high prevalence of the disease of interest with drugs of interest in common use for these diseases (e.g. HIVAIDS, malaria, etc).

• Good antenatal, labor/delivery and diagnostic services, and a willingness of staff to be trained to improve reproductive care.

• Early ANC care attendance at the clinic.

• High attendance at delivery at the health facility. (This increases the likelihood of a skilled assessment of the child at birth). OR

• A reliable plan to follow-up women delivering at home, and assess the child (e.g community health nurses/midwives to monitor home deliveries).

• Identification of a local pediatrician or doctor and commitment of such a person to review births and photographs of births and advise on management of infants. OR

• Identification, where possible, of a local specialist in the diagnosis and management of congenital anomalies.

• Identification of record-linkage systems between general out- or in-patient records and specialized clinics such as HIV clinics.

The approach described in this protocol establishes the *baseline* risk of poor birth outcomes (miscarriages, deaths, congenital anomalies, premature delivery, low birth weight) and enables calculation of *additional* risk of mortality or morbidity associated with specific diseases with or without exposure to specific drugs. Pooling data from different countries strengthens interpretation of findings beyond a single population or geographical location and enables detection of small or moderate risks in a larger population.

### Staff training

A sound obstetric, medical and drug history requires a systematic approach by health staff. A training package has been developed and includes modules to address the following:

– How to obtain informed consent, if required

– How to obtain a comprehensive medical, drug and obstetric history from pregnant women

– How to perform and record the findings of maternal physical exams

– How to encourage women to attend follow-up visits as recommended in national guidelines

– How to encourage women to deliver at the health facility

– How to capture data in the Registry database

– Procedures for tracing and following up recruited women and newborns

– How to examine a newborn infant and take a digital photograph for expert assessment (An instructional video has been developed as a tool to teach the systematic newborn surface examination).

– How and when to seek or refer a child for specialist clinical or surgical management.

### Recruitment and first antenatal visit

**Figure 1:**
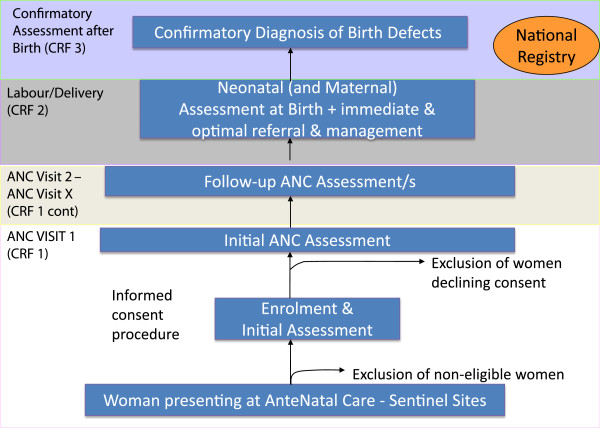
Flow diagram of study procedures.

In the African region, 18 countries report ANC attendance rates of ≥ 90% for at least one prenatal visit, with only 4 countries reporting a rate below 50% [16]. Hence, women in many parts of Africa can be enrolled at their first ANC visit as a representative sample of the pregnant population seeking care. Figure 1 provides a flow diagram of the study procedures from the initial visit at the sentinel site to the national/regional registry.

Depending upon staff capacity, attendance rates and case-load, a decision should be made on whether to enroll all women who meet the inclusion criteria or a random sample of these women (e.g. 1 day per week, or first 2 new ANC cases per day). A second decision is whether all consenting pregnant women exposed to a drug of particular current interest, or with the particular condition of interest, should be considered for enrollment irrespective of the random selection process.

At her first ANC visit, a woman’s obstetric and medical history is documented, including treatments and laboratory tests for acute or chronic conditions (e.g. diabetes, epilepsy, HIV, malaria), and referrals to higher levels of care made for specific checks as necessary. Data on routine treatments to be provided during pregnancy, including iron and folic acid, are collected and the woman is encouraged to return at scheduled ANC appointments for further checks and updated documentation on the pregnancy. She is encouraged to give birth at the healthcare facility to reduce the risk of adverse outcomes for herself and her baby, and to enable examination of the newborn by trained staff. If enrolled women do not return for ANC visits or deliver at the sentinel health facility within the expected period of time after a scheduled ANC visit or delivery date, they are followed up by phone or at home. This encourages facility attendance during the pregnancy. In the case of a home delivery it enables the pregnancy outcome to be documented. After home births, women are encouraged to return to the health facility with their infants for an examination and, if needed, medical care.

Loss to follow-up of enrollees constitutes a limitation of the Registry because it will not be known whether the participants lost have the same outcomes as those who remain under observation. Losses to follow-up can be minimised by training health staff responsible for intake to record detailed contact information of the women, counselling them about the importance of follow-up antenatal visits and delivering at the facility and providing women with a contact number at the clinic to inform nurses about any moves to travel, changes in their condition).

A final requirement is to identify a national, or preferably a local, neonatologist who can be contacted (by telephone) to advise on the management of a newborn requiring immediate clinical or surgical attention.

Participants voluntarily agreeing to participate should be enrolled at the chosen sentinel sites using the following inclusion and exclusion criteria:

### Inclusion Criteria

All of the following conditions must be met for enrolment:

• A confirmed pregnancy (by physical examination, pregnancy test or ultrasound)

• Presentation at the first antenatal visit for the current pregnancy

• Information on presence/absence of fetal abnormality unknown at the time of enrolment.

• Voluntary agreement by the woman to be followed up to term.

### Exclusion criteria

Meeting any of the following criteria would exclude a woman from enrolment:

• Refusal to participate in the Registry

• Presence of a medical, psychiatric, or social condition that interferes with the woman’s ability to provide an accurate medical or drug history or give informed consent (e.g. mentally disabled patients)

• Women who report that they will not give birth at the health facility.

A coloured, visible sticker may be placed on the ANC card of women meeting the selection criteria. The sticker signals that facility staff must fill out CRF1 (see Additional file 1) at the first visit and update it at each subsequent visit and perform a physical exam on the infant when the woman delivers.

The Registry CRF1 includes a more detailed history than standard ANC information, but otherwise all procedures routinely carried out at ANC in accordance with national guidelines are conducted without modification. It documents demographic data, medical history, obstetric history, infections, drugs and vaccine use, intermittent preventive treatments and previous birth outcomes. The CRF1 establishes whether the woman has had exposure to the medicine/s of interest, the timing of exposure and whether the exposure took place on the basis of a clinical or laboratory confirmed diagnosis. Various aids are introduced at the sentinel sites to improve women’s recall of drug exposures during pregnancy- including tablet and treatment package visual identification kits, treatment diaries or medicine storage sleeves. These recall aids are utilized at the initial and follow-up ANC assessments. Where possible, drug exposure is verified through record linkage with other systems. Results of any tests or clinical assessments are recorded. These could include voluntary counseling and testing for HIV, assessment of weight, height, gestational age, presence of chronic diseases or conditions such as diabetes, syphilis and anaemia, and pharmaceutical medications taken during or just before the current pregnancy. Table 1 provides an overview of the procedures and assessments to be conducted during each of the facility visits during a woman’s pregnancy.

**Table 1:** Review of Pregnancy Registry-related assessments and procedures conducted during antenatal and intrapartum care

	**Screening and enrollment at first visit**	**Second and subsequent visits**	**Delivery of Child**	**Post-delivery assessment**
**Antenatal Care**				
Medical & Obstetric History	X	X	X	
Physical Exam	X		X	
Counselling & assessment of concurrent illnesses (HIV, anaemia etc.)	X	X	X	
Drug exposure history	X	X	X	
Adverse outcomes in the mother	X	X	X	
**Labor/Delivery**				
Physical Assessment of infant			X	X
Adverse Events in the mother and infant			X	X

### Second and subsequent antenatal visits

At the end of each ANC visit, an appointment for the next ANC visit is made and contact details of the woman are taken and updated when necessary. Women are asked to return for follow-up assessments as normally scheduled, generally at least four ANC visits for each pregnancy. Updated information on the woman’s obstetric and medical conditions, medication use, smoking and alcohol consumption are also obtained during follow-up ANC visits (CRF 1). At each ANC visit the woman is counseled to return to the facility for delivery.

### Labor and Delivery

The labor/delivery ward staff identifies Registry enrollees by the brightly coloured labels on the ANC cards. Immediately after delivery, the baby (whether alive or stillborn) will undergo a careful physical surface examination and the details recorded on a Pregnancy Outcome CRF (CRF2 – see Additional file 2). This form also records information on any abortions or miscarriages which terminate the pregnancy. The vital status of the infant is recorded as well as sex, head circumference, length and weight. The date, place and time of examination and the age of the baby at examination (number of days since birth) is documented on CRF2 , so that analysis can separate babies assessed at birth from those assessed some time later. Any abnormalities observed and any adverse outcomes in the mother or baby are noted and reported in the CRF.

**Figure 2:**
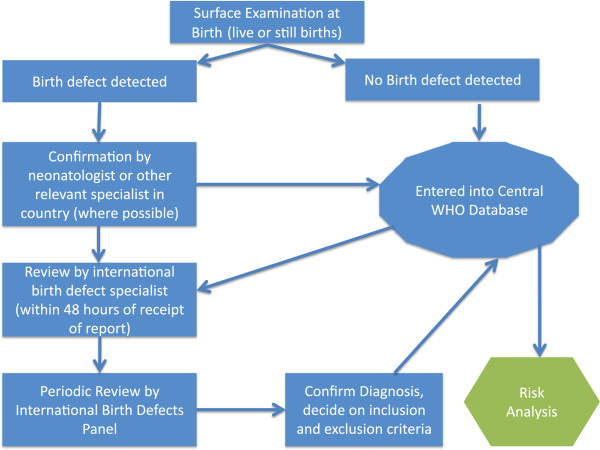
Assessment process for congenital anomalies.

Training materials have been developed to support antenatal facility staff and delivery staff, to complete the CRFs and to assess a newborn. The systematic surface examination, as outlined in the training video, identifies congenital anomalies which are visible to the examiner. Some of these could be of major surgical or clinical importance (refer to the section below on ascertainment and classification of outcome). Digital photographs of any abnormality are taken for the purposes of confirmation and classification by an international Birth Defect Panel. For the process of confirming birth defects, see the section on ascertainment of outcome and Figure 2.

Photographs will not be taken if a mother refuses to have a photograph taken of her infant at the time of birth even if she had agreed to do so earlier. Her decision is recorded on CRF2. Infants with major anomalies are referred to a specialist for confirmation of the diagnosis (see CRF 3 – Additional file 3) and further management.

It is not expected that all staff performing deliveries in resource-limited settings, usually nurses/midwives, can auscultate for heart murmurs nor is it assumed that assessment of other internal problems through ultrasound equipment will be routinely available. The Registry does not require that the palate be examined for oral clefts nor that the hips be examined for congenital hip dysplasia. Consequently, these important malformations will not be detected unless clinical signs are noted and further tests are performed.

If a woman does not deliver at the facility, arrangements need to be made to carry out the surface examination as soon as possible after birth. In resource-limited settings, where home-based delivery rates can be high and referral systems inefficient, completion of the initial examination and follow-up on findings within 12 weeks is considered acceptable, especially if the examination is done at a facility. A community health nurse can be taught to conduct home assessments for women who do not come to the facility to deliver. Community-based staff would need to be adequately trained and equipped to ensure that off-site assessments are equivalent to facility-based assessments, especially if many assessments are likely to be home-based. Although home-based follow-up is important, the high rates of facility-based BCG vaccinations even in the poorest areas of Africa, suggests that a facility assessment can be undertaken at the time of the first vaccination. Losses to follow-up and un-assessed or uninvestigated neonatal deaths or birth defects represent a limitation, of the Registry as the possibility of infanticide or neglect could occur if the newborn has a visible abnormality such as clubfoot, cleft lip or myelomeningocele [17].

### Ethics

The WHO Ethics Review Committee and the ethics committees of the 5 participating countries (Brazil, Ghana, Kenya, Tanzania, and Uganda) implementing the pilot study have reviewed and approved the protocol for use. Any site or country wanting to implement the protocol can do so and establish whether local/national ethics approval is required.

Investigators, in consultation with public health officials and ethics review committees, should determine whether the Registry will be required to take informed consent from enrollees and, if so, whether written or verbal consent is appropriate.

Some ethics committees may waive the need for written informed consent on the basis that:

i) the research is observational, part of standard of care and involves no additional risk;

ii) a waiver does not adversely affect the rights and welfare of the subjects;

AND

iii) the subjects will be provided with pertinent information, if appropriate, after analysis [18].

In settings where the Registry is seen as part of a government program evaluating treatment policy for high priority diseases in the country, a waiver may be sought since written informed consent increases the time spent with each woman at ANC and can reduce time for patient care.

Participation in the Registry is voluntary. Hence, if a woman refuses to be part of the Registry, she can do so without consequence to her medical care. If a woman refuses to have a digital photograph taken of her infant (i.e. in the case of a malformation), her data may be included without photographs, with her agreement.

The woman must be assured that all information is kept confidential and that her identity will be anonymized when data are analyzed. A guidance note is provided to women on the safe use of medicines in pregnancy in order to engender confidence in the medicines being prescribed for her during her pregnancy and the need for exercising caution when using over-the-counter and other un-prescribed medicines (Additional file 4).

Clinical staff at participating health facilities are trained to rapidly identify and refer infants with adverse birth outcomes or birth defects to the appropriate medical providers or healthcare facilities, to improve neonatal outcomes. However, each participating facility must decide how to cover referral and treatment costs.

All significant findings from analyses need to be communicated to sentinel site staff on an ongoing basis. Should any information come to light, which may directly or indirectly benefit or harm the patient, the attending clinician or health care provider should be informed as soon as possible (e.g. if a baby is born with a hereditary or potentially preventable birth defect the mother may need counselling, or if it is noticed that a clinic is prescribing inappropriate medication to pregnant women). The woman should be informed that anonymized information from all countries will be pooled in a relational online database and the findings shared with the facility.

### Ascertainment and classification of outcome

An International Birth Defects Assessment Panel has been established by WHO to review, confirm and classify major birth defects reported by the participating sites. Although all birth defects are documented, exclusion criteria have been developed by the Panel on the categories of birth defects which are not relevant. All defects that do not constitute “major malformations” defined as a structural abnormality with surgical, medical or cosmetic importance and hereditary and chromosomal disorders that are not caused by exposure to drugs, are excluded.

The following features/anomalies are therefore excluded from the primary endpoint of assessing drug-related risk [19]:

• minor anomalies ( e.g. transverse palmar crease)

• normal variations (e.g. umbilical hernia in African infants)

• hereditary disorders (e.g. polydactyly, postaxial Type B)

• birth marks (e.g. haemangiomas; congenital moles)

• chromosome abnormalities (e.g. Down syndrome)

• positional deformities (e.g. congenital hip dislocation in infants who are born in breech position;

• features of prematurity (e.g. undescended testes and patent ductus arteriosus in infants less than 37 weeks gestational age;

• biochemical abnormalities(e.g. carriers of cystic fibrosis, and abnormal haemoglobins that may be identified during newborn screening.

### Monitoring and quality control

Progress and training of staff needs to be monitored regularly for quality and reliability of procedures, examinations and data. This will reduce the possibility of biases in enrolment and the risk of false negative results (e.g. when a surface examination of a baby was not complete). Detection of the presence/absence of malformations depends upon the training, skill, and experience of the assessor. Absence of malformations will not be reassuring, nor will data be reliable if assessors have not looked for them or are unable to assess a child. Only reliable data should contribute to national policy. For this reason it is recommended that a random sample of 5% of all pregnancy outcomes be re-evaluated by a second nurse and the comparison of these separate findings be recorded.

### Analytic approach

The cohort study design allows for analysis of the data using three approaches – 1) as a complete cohort, 2) using a case–control approach and 3) using a case-cohort approach. The cohort analysis facilitates examination of the demographic and clinical information about the mothers and infants and prevalence of risk factors such as drug exposures during a particular trimester. Case–control studies can be performed to determine the risk of specifically defined birth defect/s (e.g. neural tube defects) and whether or not these are associated with in-utero exposure to particular medicines. A case-cohort analysis approach can be used to test hypotheses not initially considered when the cohort was initiated or to analyze data where additional assessments have been carried out. Using this approach, a randomly selected sub-cohort of non-cases will be selected from the main cohort and used to compare risk factors for any or all adverse outcomes.

Case–control studies enable the assessment of whether exposures to specific drugs are associated with an increased frequency of specific, major malformations. The protocol enables assessment of the additional risk of adverse outcomes consequential to drug exposure.

Confounding is addressed through the use of multivariate analysis, where the sample size is adequate. Alternatively, cases and controls can be matched in order to address the potential for confounding. Subsequent analyses will therefore use paired statistical methods. In the uncommon situation where the underlying condition being treated and the suspected drug/s give rise to the same adverse birth outcome or birth defect, the issue of confounding by indication arises. In such instances, assessing the differences in risk profile for different drugs used to treat the same condition could assist in addressing this potential confounding.

At the point of analysis, for both the exposed and the unexposed comparison group, the pre-defined inclusion/exclusion criteria for major congenital anomalies need to be used to specify which features count as major malformations. To include minor features as “major” abnormalities unfairly and inappropriately exaggerates the apparent fetal effects of an exposure [20]. This makes it essential that studies of potential teratogens set inclusion/exclusion criteria in advance of analysis to evaluate the test group. The same criteria must be used to evaluate their unexposed comparison group. Therefore, inclusions and exclusions are not conducted at sentinel sites but by the International Birth Defects Panel of experts, without prior knowledge of group/exposure. Sentinel sites are encouraged to report all anomalies (major and minor) and all birth outcomes regardless of the type or severity of such anomalies, whereas the Birth Defects Panel adjudicates the defect as major or minor.

The background risk of major congenital anomalies and other adverse birth outcomes (e.g. stillbirths/miscarriage/abortion) at sites is estimated from the proportion of major anomalies and other adverse outcomes identified in newborns of women who report no inter-current clinical condition or exposure to potentially teratogenic medication during the relevant stage of pregnancy. Analyses should be carried out for drug exposures in all three trimesters as well as for the first trimester only. As the number of enrolled women increases over time, sub-analyses that assess the safety of individual medicines or combination therapies becomes possible, as well as detailed analysis based on dose and duration of exposure.

Descriptive statistics are the primary approach for summarizing data from pregnancy exposure registries with frequencies of outcomes expressed via absolute risk, relative risk, and population attributable risk, with 95% confidence intervals. Data collected through the Registry should be evaluated with standard periodic reviews of the database to assess the primary and secondary endpoints as well as to assess the quality of the information submitted by contributing sites based on pre-defined indicators (e.g. missing data fields, inconsistencies between fields). Statistical analysis should consider heterogeneity within the data set in terms of methods employed, quality of the data and other site-specific differences. If different methods of assessing drug exposure are used, analysis of outcomes should be stratified by method (e.g. ANC card, prescription, record linkage or self report), as some are more precise than others.

### Sample Size Calculations

The estimated sample size depends on the strength of the teratogenic effect, the background frequency of adverse pregnancy outcomes in the population of interest as well as the number expected to be exposed to the drugs under study, and the choice of comparator group (background rate, or contemporaneous internal comparator) [21]. Much of this information is unknown in the proposed settings for such a registry [22]. Therefore, sample size should be finalized after the potential for harm of a particular substance is characterized by an initial cohort of recruited women.

The background incidence of congenital anomalies is established through a contemporaneous internal comparator group. Table 2 below describes two options for estimating sample size based on case/comparators ratio: 1 case/ 1 comparator and 1 case/ 4 comparators. Two options are also presented regarding the relative risk to be detected (i.e. 2 or 10). Three different incidences of risks are chosen in the comparator group. They correspond to a baseline incidence of major malformations of 1% or 5%, and an incidence of 0.1% (which is generally accepted incidence of the more common malformations) [21]. A continuity correction of 10% for the chi-squared test has been included in the estimation.

**Table 2:** Sample size estimations based on background incidence, case/comparator ratio and anticipated relative risk including continuity correction

	**1 case / 1 comparator**				**1 case / 4 comparators**			
	**RR to be detected : 2**		**RR to be detected : 10**		**RR to be detected : 2**		**RR to be detected : 10**	
Incidence in comparator group	Cases	Comparators	Cases	Comparators	Cases	Comparators	Cases	Comparators
5%	474	474	19	19	274	1096	10	40
1%	2515	2515	121	121	1445	5780	61	244
0.1%	25471	25471	1272	1272	14621	58484	628	2512

Based on the calculations provided in Table 2, it can be estimated that a cohort of at least 2515 exposed women per drug of interest in the 1^st^ trimester and an equal number of comparison women would achieve a power of 80 % to detect a doubling of risk of major birth defects for any given drug of interest, assuming a background incidence of birth defects of 1% (excluding congenital anomalies, cardiovascular malformations and genetic disorders which may not be detected) and a significance level of P < 0.05. The sample size was calculation was calculated using WinPepi version 11.4 with continuity correction.

### Data management

The WHO Pregnancy Registry database has been developed using the free access software OpenClinica. The database has been designed to accommodate electronic and paper-based CRFs depending on the preferences of the contributing sites.

Data entered on paper CRFs by health staff can be subsequently entered into an electronic database. Investigators have experience with direct data entry into the online OpenClinica database and with offline EPIdata databases, with periodic transfer of both into a common statistical package. The EPIdata programme is also an open access standalone database but does not have an audit trail. This can be provided to sites not requiring an audit-trail and agreeing to routinely download the data into the pooled Registry database. This multi-pronged approach for data capture is intended to facilitate data capture at sites where online connectivity and feasibility of electronic data capture approaches may be limited, while ensuring ease of electronic data capture where such infrastructure exists.

Future steps envisaged are to ensure that CRFs are adapted for data capture using mobile phones and other hand held electronic devices. Depending on the approach adopted by the site, systems can be supported at the sites to ensure the integrity of the data capture and contributions to a pooled Registry database.

Pre-defined searches, reports and analyses from the pooled Registry will be developed in collaboration with sites and countries in order to facilitate routine analysis of data by sites, countries and internationally. Pre-defined analyses for determination of drug effect will be agreed by contributing sites and investigators before publication. National coordinators and research sites agreeing to pool data into the database can be trained to import data from the database into statistical packages such as Stata and SPSS.

### Feedback and communication

The Registry is an opportunity to build trust and confidence in the communities it serves. ANC staff should inform communities that they will be monitoring safety of treatments during pregnancy. This will reduce the likelihood of rumors and allay fears.

Through the automated online login system, pre-designed tables and reports can be generated to provide indicators of maternal and child health to the relevant authorities and interest groups on a regular basis. Similarly, routine progress reports by the registry investigators to the facility staff can ensure ongoing feedback.

## Discussion

A Pregnancy Registry is critical when the extent of human reproductive risk remains unknown for an effective medication, thus either excluding an important group from treatment, or increasing their risk. In important diseases – HIV and malaria as examples– the potential for inadvertent exposure of the fetus to treatment is high and may occur during early organogenesis, when women may not be aware that they are pregnant. In Sub-Saharan Africa, where 68% of the world’s HIV population reside and approximately 25 million pregnant women are at risk of *P falciparum* infection [23,24], the major components of first line treatments for malaria and HIV - artemisinins and efavirenz - are contraindicated in the 1^st^ trimester. Termination of pregnancy is not a legal or cultural option in many African countries, and hence exposure of pregnant women to medications that may increase the risk of birth defects could cause patient anxiety or illegal induced termination of a pregnancy. When exposure has not occurred, a pragmatic approach in pregnancy involves either use of a less effective treatment drug or, in the case of HIV, a regimen change which may be more costly.

Recent modeling studies projecting the clinical benefits and risks of using EFV versus NVP to women of childbearing age suggest that the survival gains through use of EFV-based initial regimens in HIV-positive US women of childbearing age would be small [25]. However, in Sub-Saharan Africa, modelling determines that the survival gain of using EFV instead of NVP would substantially outweigh the additional number of birth defects [26]. This is based on current nevirapine and EFV toxicity data and birth defect rates from the Antiretroviral Pregnancy Registry and excludes benefits from the prevention of transmission to infants. WHO guidelines for PMTCT recognize that while data rule out a 10-fold or higher increase in risk with first-trimester EFV exposure, it will take time to establish safety using large numbers because even an elevated risk is likely to be less than 1% [6].

Pregnancy registries minimize the penalties of limited evidence by obtaining systematic, reliable data on whether important drugs are teratogenic by monitoring exposure in large enough numbers to provide levels of confidence regarding hazard or benefit. This WHO Pregnancy Registry protocol for assessing the risk of teratogenicity of medicines in pregnancy has been piloted in five developing countries: Brazil, Ghana, Kenya, Tanzania and Uganda; testing is still ongoing in Brazil and beginning elsewhere. The approach has proven to be simple and feasible in typical resource-constrained settings. The methods are pragmatic and have been devised to support and improve maternal and neonatal care, provide immediate benefit to pregnant women, and complement and build capacity of reproductive health staff to provide better care – all of which increase the sustainability of the approach. The generalizability of the methods across countries enables data to be pooled.

There are three important features of this WHO Pregnancy Registry protocol which stand out from most other registries. The first is the simplicity of including women agreeing to take part at their first facility visit for care during the pregnancy. This not only represents the population of pregnant women coming for care but also enables later comparison of birth defects among women who have been exposed to a medicine with those who have not. The second feature is the generic applicability of the approach irrespective of drug or disease, and the third is improvement of staff capacity to manage and monitor pregnancies and newborns. These qualities add to the practicality and cost-effectiveness of the protocol.

In developed countries there are systems in place to monitor pregnancies and their outcomes. These data provide a population that can be compared with birth defect prevalence in cohorts exposed to specific drugs [27]. In the United States, a prevalence of 1.5-2.5% major birth defects at birth has been reported [28,29], a rate which includes heart defects, hip dysplasia and cleft palate. In resource-poor countries, population data on maternal outcomes and malformations are very limited [11]. Hence, determination of any additional risk associated with drug exposure in developing countries requires collection of data on the baseline risk of birth defects in the same population. By enrolling women as they come to ANC care and assigning them to the exposed or unexposed population at the end of pregnancy, the assessment of the prevalence of birth defects in a disease-negative, unexposed population can be determined and becomes directly comparable with prevalence of birth defects in a disease-positive, treated population.

A second benefit is that this registry provides information on many medications and health conditions simultaneously; its value and sustainability exceed the timeframe of safety-assessment for a specific drug. Not only can teratogenic potential of the disease or maternal condition (e.g. HIV, epilepsy, dengue) itself be determined [30,31], but the disease plus treatment effects in the same population can be evaluated. Importantly, the data can be augmented over time by increasing the number of contributing countries and sentinel sites adding data on the effect size in different populations, and strengthening levels of confidence in the results. Indeed, the power of the approach lies in its broad application to a variety of settings in which women may have more than one infectious disease or condition during the course of the pregnancy and may also have been exposed to many drugs.

A third benefit is capacity building for improvement of reproductive health. Although the objective of the WHO Pregnancy Registry is to assess the safety of medicines to which pregnant women are exposed, its prospective nature and the requirement for any sentinel site contributing data to have or create an ANC program that meets certain maternal and neonatal care standards, strengthens its value for monitoring and managing these important outcomes. In the optimal Registry scenario, women come early to ANC in pregnancy, diagnostic capacity for diseases is linked to records at the ANC clinic, ANC, perinatal and postnatal records are linked and precise data on treatment exposure are available, and all deliveries occur at the facility. The Registry training modules and protocol actively supports these features. Thus, the protocol and its implementation represent a novel way of monitoring reproductive health, collecting important indicators of maternal and neonatal health and service delivery and providing an opportunity to build capacity for maternal and child healthcare through supporting the development of diagnostic skills for congenital anomalies and the creation of teratology information services at different institutions in resource-poor settings.

As with all observational studies, this Pregnancy Registry is subject to several limitations. Key challenges and limitations are poor data quality or poor medical and drug histories arising from lapses in memory (particularly due to late presentation to the first ANC visit) and the inadequacy of existing medical records to confirm reported exposures to medicines – both of which limit precision of whether and when drug exposure might have occurred. A second limitation is the potential for losses to follow-up because of home births – measures to minimize this problem have been described earlier. It is possible that higher losses occur for riskier pregnancies.

Any registry that requires consenting women to take part has potential for selection bias; women consenting might have different characteristics from those who do not consent to take part [32]. A fourth limitation is that where there is a high case load and a number of babies are being examined concurrently, it is possible that even major birth defects are missed, if the baby is not examined systematically. Lack of local expertise and limited diagnostic capacity (e.g. chromosome analysis) at each of the sites (and sometimes even nationally) precludes the assessment of infants whose diagnosis cannot be assessed from the review of photographs. Finally, heterogeneity of data quality, and the presence of unknown confounders may pose challenges in the analysis of data and the attribution of cause.

This protocol is not reliable or informative about early pregnancy loss due to specific drug exposure. This is because only women with viable pregnancies come for antenatal care (the timing of first antenatal visit is later in Sub-Saharan Africa than in other regions [33]), and because an acute infection (e.g. malaria) as well as other factors can cause pregnancy losses. The Registry is designed to detect only anomalies visible from surface examinations at birth. Birth defects arising from nutritional deficiencies or genetic factors that affect neural-development and internal organ systems are unlikely to be detected; further elaboration of this study design and access to specific skills and equipment often available at a tertiary facility would be required for these purposes.

Finally, communication strategies around the safety profile of medicines in pregnancy would need to reduce potential for heightened fear and rumours around particular drugs or conditions. Efforts should be made to communicate effectively with women providing factual information, particularly in settings where exposure to the drug with limited information on safety has already occurred.

This protocol is supported by training materials that have been tested in four African countries and in Brazil. These are available on request from the corresponding author to those intending to initiate a Pregnancy Registry, particularly with a data-pooling agreement with WHO already in place enabling the country to benefit from the data it collects, as well as from an international effort. The Pregnancy Registry that involves systematic, improved care and assessment of the woman during pregnancy and her newborn at birth also provides a robust source of evidence of the safety of medicines used in pregnancy.

## Abbreviations

ACTs: Artemisinin-based Combination Therapies; AIDS: Acquired Immune Deficiency Syndrome; ANC: Antenatal clinic; ARV: Antiretroviral; CRF: Case record form; EFV: Efavirenz; HIV: Human Immunodeficiency Virus; NVP: Niverapine; PMTCT: Prevention of Mother to Child Transmission; WHO: World Health Organization.

## Competing interests

The authors declare that they have no competing interests.

## Authors' contributions

UM and MG drafted the protocol with input from all the other co-authors. LBH was involved in the development of the methodology and the inclusion and exclusion criteria for the physical features of the infants born to enrolled mothers. FS, EE and VM provided critical advice on the design of the protocol. MY, ES, EA and CC were involved in the detailed development and refinement of the protocol and case record forms during the pilot period. JS provided support and advice relating to data management and MP and EE provided biostatistical support. All authors read and approved the final manuscript.

## Authors' information

UM has been involved in the development of regulatory and programmatic pharmacovigilance systems in resource-limited settings. She served as scientific co-ordinator for the pregnancy registry pilot project. CC is a lecturer and Acting Head of the Department of Epidemiology and Disease Control of the School of Public Health, University of Ghana. Her research interests include epidemiology, control of infectious diseases and maternal and child health issues. EA is a pharmacist and clinical trials manager with a research interest in the validity of pharmacoepidemiological participant-reported outcomes. MY served as the study co-ordinator for the pregnancy registry pilot study. She helped establish birth defect surveillance systems in hospitals in Ghana and is currently studying medicine at Stanford University. ES is Professor of Pharmacology and Scientific Director at the Faculty of Medicine of Eduardo Mondlane University, Mozambique. She is a Senior Research Fellow at Manhiça Foundation with interests in pharmacovigilance and use of drugs in pregnancy. JS served as the data manager for the WHO pregnancy registry pilot study and has been involved in this capacity in malaria, TB/HIV and pregnancy-related studies since 2005. MP is professor and head of the Unit for Applied Biostatistics at the medical faculty at University of Gothenburg. He has been involved in several medical drug studies including intermittent treatment of malaria during infancy for the WHO. MP has also been responsible for several large register based studies on adverse drug reactions in the Nordic countries. VM is a specialist in international public health with a primary expertise in maternal, child, adolescent health and nutrition. At WHO she is responsible for the integration and partnerships work of the Department of Reproductive Health with HIV, Malaria programs and other relevant partners. Since 2011 she is the co-chair of the Roll Back Malaria-Malaria in Pregnancy Working Group. EE is a teratologist, medical embryologist and head of the European Network Teratology Information Services (ENTIS) at the Centre de Référence sur les Agents Téeratogènes, Hopital Trousseau, Paris. The Centre, led by EE, has over 30 years informed health professionals about the risk of teratogenicity and medication induced fetal toxicity during pregnancy, and assists knowledge & management of such risks. FMS conducted research for many years in the University of London on mechanisms of developmental toxicity. He was also the founding Director of the UK National Teratology Information Service. LBH is the Director of the North American AED (antiepileptic drug) Pregnancy Registry in Boston since 1997. He is professor of paediatrics at Harvard Medical School and chief of the Genetics and Teratology Unit, Pediatric Services, Massachusetts General Hospital, Boston. MG is the Malaria Leader of The UNICEF/UNDP/World Bank/WHO Special Programme for Research & Training in Tropical Diseases. She is an epidemiologist with expertise in drug development and is responsible for regular assessments of antimalarial drug safety in WHO and contributes to a WHO-wide collaboration assessing drug safety in pregnancy in resource-poor settings.

## Pre-publication history

The pre-publication history for this paper can be accessed here:

http://www.biomedcentral.com/1471-2393/12/89/prepub

## Supplementary Material

Additional file 1 **Case Record Form 1. **Antenatal data sheet. Data capture form used at initial recruitment and during antenatal follow-up visits. Click here for file

Additional file 2 **Case Record Form 2. **Pregnancy Outcome Sheet Data capture form used during labor/delivery period or during assessment of mother and infant within 12 weeks after birth. Click here for file

Additional file 3 **Case Record Form 3. **Data capture form used during the confirmatory assessment of the infant for the suspected birth defect. To be completed by a relevant medical specialist (e.g. neonatologist, teratologist). Click here for file

Additional file 4 **Guidance on the use of medicines during pregnancy. **A note given to recruited women on the prudent use of medicines during the course of the pregnancy. Click here for file

## References

[B1] Ministers of Foreign Affairs of Brazil. France, Indonesia, Norway, Senegal, South Africa, and Thailand Oslo Ministerial Declaration--global health: a pressing foreign policy issue of our time Lancet 2007 369 9570 1373 1378 10.1016/S0140-6736(07)60498-X 17448824

[B2] WHO Assessment of the safety of artemisinin compounds in pregnancy: report of two joint informal consultations convened in 2006 2007 WHO, France http://www.who.int/malaria/publications/atoz/9789241596114/en/

[B3] WHO Assessment of the safety of artemisinin compounds in pregnancy. Geneva: Report of two informal consultations convened by WHO in 2002 2003 WHO, Geneva http://www.who.int/malaria/publications/atoz/whocdsmal20031094/en/index.html

[B4] Clark RL Embryotoxicity of the artemisinin antimalarials and potential consequences for use in women in the first trimester Reprod Toxicol 2009 28 3 285 296 10.1016/j.reprotox.2009.05.002 19447170

[B5] Li Q Weina PJ Severe embryotoxicity of artemisinin derivatives in experimental animals, but possibly safe in pregnant women Molecules 2009 15 1 40 57 10.3390/molecules15010040 20110870 PMC6256922

[B6] Ford N Calmy A Mofenson L Safety of efavirenz in the first trimester of pregnancy: an updated systematic review and meta-analysis AIDS 2011 25 18 2301 2304 10.1097/QAD.0b013e32834cdb71 21918421

[B7] Siberry GK Williams PL Mendez H Seage GR 3rd Jacobson DL Rich KC Griner R Tassiopoulos K Kacanek D Mofenson LM Miller T Dimeglio LA Watts DH For the Pediatric HIVAIDS Cohort Study (PHACS). Safety of tenofovir use during pregnancy: early growth outcomes in HIV-exposed uninfected infants. AIDS 2012 26 9 1151 1159 10.1097/QAD.0b013e328352d135 22382151 PMC3476702

[B8] WHO Guidelines for the treatment of malaria 2010 Second WHO, Geneva http://www.who.int/malaria/publications/atoz/9789241547925/en/index.html

[B9] WHO Antiretroviral drugs for treating pregnant women and preventing HIV infection in infants: recommendations for a public health approach 2010 WHO, Geneva 2010 version 26180894

[B10] European Collaborative Network Factors associated with HIV RNA levels in pregnant women on non-suppressive highly active antiretroviral therapy at conception Antivir Ther 2010 15 41 49 20167990 10.3851/IMP1489 PMC3428879

[B11] Myer L Carter RJ Katyal M Toro P El-Sadr WM Abrams EJ Impact of antiretroviral therapy on incidence of pregnancy among HIV-infected women in Sub-Saharan Africa: a cohort study PLoS Med 2010 7 2 e1000229 10.1371/journal.pmed.1000229 PMC2817715 20161723

[B12] WHO HIV/AIDS Programme Programmatic Update: Use of antiretroviral drugs for treating pregnant women and preventing HIV infection in infants 2012 WHO, Geneva http://www.who.int/hiv/pub/mtct/programmatic_update2012/en/index.html

[B13] Sevene E Bardají A Mariano A Machevo S Ayala E Sigaúque B Aponte JJ Carné X Alonso PL Menendez C Drug exposure and pregnancy outcome in Mozambique Paediatr Drugs 2012 14 1 43 49 10.2165/11591270-000000000-00000 22145781

[B14] FDA Guidance for Industry: Establishing Pregnancy Exposure Registries. US Department of Health and Human Services, Food and Drug Administration, Center for Drug Evaluation and Research (CDER), Center for Biologics Evaluation and Research (CBER) 2002 http://www.fda.gov/cber/gdlns/pregexp.pdf

[B15] Dellicour S Ter Kuile FO Stergachis A Pregnancy Exposure Registries for Assessing Antimalarial Drug Safety in Pregnancy in Malaria-Endemic Countries PLoS Med 2008 5 e187 10.1371/journal.pmed.0050187 18788893 PMC2531138

[B16] Assessing Progress in Africa toward the Millennium Development Goals MDG Report 2010 http://www.africa-union.org/root/au/Conferences/2010/september/mdg/English%20MDGs%20report.pdf

[B17] Baiden F Hodgson A Adjuik M Adongo P Ayaga B Binka F Trend and causes of neonatal mortality in the Kassena-Nankana district of northern Ghana, 1995–2002 Trop Med and Int Health 2006 11 4 532 539 10.1111/j.1365-3156.2006.01582.x 16553937

[B18] Council for International Organizations for Medical Sciences International Ethical Guidelines for Biomedical Research Involving Human Subjects 1993 CIOMS, Geneva http://www.codex.uu.se/texts/international.html

[B19] Holmes LB Westgate MN Inclusion and exclusion criteria for malformations in newborn infants exposed to potential teratogens Birth Defects Res A Clin Mol Teratol 2011 91 9 807 812 10.1002/bdra.20842 21800414

[B20] Holmes LB Baldwin CR Smith E Habecker L Glassman SL Wong SL Increased frequency of isolated cleft palate in infants exposed to lamotrigine during pregnancy Neurology 2008 70 2152 2158 10.1212/01.wnl.0000304343.45104.d6 18448870

[B21] Wyszynski DF Pregnancy exposure registries: Academic opportunities and industry responsibility Birth Defects Research Part A: Clinical and Molecular Teratology 2009 85 1 93 101 10.1002/bdra.20525 19107953

[B22] Christianson A Howson CP Modell B March of Dimes: Global report on birth defects. The hidden toll of dying and disabled children. March of Dimes Birth Defects Foundation 2006 March of Dimes Birth Defects Foundation, White Plains, New York

[B23] UNAIDS World AIDS Day report 2011 UNAIDS, Geneva

[B24] Desai A ter Kuile F Nosten F McGready R Asamoa K Brabin B Epidemiology and burden of malaria in pregnancy Lancet 2007 7 93 104 10.1016/S1473-3099(07)70021-X 17251080

[B25] Hsu HE Rydzak CE Cotich KL Wang B Sax PE Losina E Quantifying the risks and benefits of efavirenz use in HIV-infected women of childbearing age in the USA HIV Medicine 2011 12 2 97 108 10.1111/j.1468-1293.2010.00856.x 20561082 PMC3010302

[B26] Ouattara EN Anglaret X Wong AY Chu J Hsu HE Danel C Projecting the clinical benefits and risks of using efavirenz-containing antiretroviral therapy regimens in women of childbearing age AIDS 2012 26 5 625 634 10.1097/QAD.0b013e328350fbfb 22398569 PMC3834615

[B27] FDA List of pregnancy exposure registrieshttp://www.fda.gov/scienceresearch/specialtopics/womenshealthresearch/ucm134848.htm#conditions

[B28] Nelson K Holmes LB Malformations due to presumed spontaneous mutations in newborn infants New Engl J Med 1989 320 19 23 10.1056/NEJM198901053200104 2909875

[B29] Peller AJ Westgate MN Holmes LB Trends in congenital malformations, 1974–1999: effect of prenatal diagnosis and elective termination Obstet and Gyn 2004 104 5 pt 1 957 964 10.1097/01.AOG.0000142718.53380.8f 15516385

[B30] Sharma JB Gulati N Potential relationship between dengue fever and neural tube defects in a northern district of India Int J Gynaecol Obstet 1992 39 4 291 295 10.1016/0020-7292(92)90260-P 1361462

[B31] Xiao KZ Zhang ZY Su YM Liu FQ Yan ZZ Jiang ZQ Central nervous system congenital malformations, especially neural tube defects in 29 provinces, metropolitan cities and autonomous regions of China: Chinese Birth Defects Monitoring Program Int J Epidemiol 1990 19 4 978 982 2084031 10.1093/ije/19.4.978

[B32] Holmes LB Baldwin EJ Smith CR Increased frequency of isolated cleft palate in infants exposed to lamotrigine during pregnancy Neurology 2008 70 2152 2158 10.1212/01.wnl.0000304343.45104.d6 18448870

[B33] AbouZahr CL Wardlaw TM Antenatal care in developing countries: promises, achievements and missed opportunities: an analysis of trends, levels and differentials, 1990–2001 2003 WHO, Geneva

