# Supplementary material for: Protocol for a drugs exposure pregnancy registry for implementation in resource-limited settings
Source: BMC Pregnancy Childbirth. 2012 Sep 3;12:89. doi: 10.1186/1471-2393-12-89 (PMC3500715; doi:10.1186/1471-2393-12-89)
Supplement: Additional file 1 — Case Record Form 1. Antenatal data sheet. Data capture form used at initial recruitment and during antenatal follow-up visits. [file 1471-2393-12-89-S1.pdf]

World Health Organization

PREGNANCY REGISTRY PILOT STUDY

Subject's initials: ________________

Clinic name: ________________________

Clinic ID: ________________________

Registry ID: ________________________

**Informed consent date**		
**First date of last menstrual period**	________ dd mm yy or ________ how long ago or NK	
**Expected date of delivery**	________ dd mm yy	
**Actual date of delivery**	________ dd mm yy	
	Scheduled Visit Dates	Actual Visit Dates
**Visit 1**		
**Visit 2**		
**Visit 3**		
**Visit 4**		
**Visit 5**		
**Visit 6**		
**Visit 7**		

**Pregnancy registry**	**Ante-natal data-sheet**<br>Clinic______________________	Registry ID:<br>Clinic ID:

Visit 1			
Visit date: ___________ dd mmm yy	Assessor's name: _______________	Assessor's title/position: _________	Assessor's signature: ____________
Age: _______ years	Height: __________ cm	Weight: _________ kg	Mid upper arm circumference: ____________ cm
Fundal height: _______ cm NA	Fetal heart sound? Yes No NA	Ultrasound? Yes *(complete 'tests')* No	

Gravida (Total number of pregnancies, including the current pregnancy): _______	Para (Number of liveborn and stillborn children): _______								
How many of your children born alive have died?	NA	0	1	2	3	4	5	6	7+
How many previous multiple pregnancies have you had?	NA	0	1	2	3	4	5	6	7+
How many stillborn children have you had?	NA	0	1	2	3	4	5	6	7+
How many miscarriages or abortions have you had?	NA	0	1	2	3	4	5	6	7+
Are you related by birth to the father of this child? Yes, how? ______________________ No NK									
Were any of your babies born with deformities? NA No Yes (please provide details for each baby if known):									
Were you or any of your family members born with deformities? No Yes<br>If **yes**, what is your relationship to this person: self mother father sibling child's father<br>Description:									

Do you smoke?	Do you drink alcohol?	Do you use illegal drugs?
Yes (cigarettes/day?) No	Yes (drinks/week?) No	Yes *(complete "treatments")* No NK

I will now ask you about your health. Please tell me about treatments you have taken **<u>during (and just before) your pregnancy</u>**. Please include all treatments you have taken, even if you think they are not related to your pregnancy. Please consider anything a health worker, traditional healer, birth attendant, shop-keeper, relative or friend has given or sold you. Reporting treatments will not harm or cause trouble to you or anyone else.

**Have you had any of the following medical problems?** *(Circle any condition reported by the participant or circle 'None'. If participant reports an illness, record details in 'medical history' section opposite.):*										
Epilepsy	Genital Herpes	TB	Gonorrhoea	High blood pressure	Diabetes	Rubella	HIV	Syphilis	Other infections	None
**If you have had any of these medical problems, have you taken any treatments for them?** *(If yes, record treatments reported in "treatments" section opposite):* Yes No NA										

During this pregnancy, or within a month before you became pregnant, have you:
(For questions below, write any conditions, treatments, and tests in the "medical history", "treatments", "test results" section opposite.)

Had malaria?	Yes	No	NK
Taken any treatments to prevent malaria?	Yes	No	NK
Had any fever other than malaria?	Yes	No	NK
Had any vaccines?	Yes	No	NK
Had any blood transfusions?	Yes	No	NK
Had any other injections?	Yes	No	NK
Had any vaginal bleeding?	Yes	No	NK
Had any other condition (apart from those mentioned above)?	Yes	No	NK
Have you taken any traditional or herbal medicines?	Yes	No	NK
Taken any other treatment, including routine treatments (e.g. folic acid, iron supplements, multivitamins, deworming tablets)?	Yes	No	NK
Had any tests at a clinic (apart from an ultrasound)?	Yes	No	NK

For midwife to complete:
(For questions below, write any treatments and tests prescribed in "medical history" and "test results" section opposite)

At the clinic today, will this patient be prescribed one or more treatments?	Yes	No
At the clinic today, will this patient be given or prescribed one or more tests?	Yes	No

Visit 2			Registry ID	Clinic ID
Visit date: ________ dd mmm yy	Assessor's name: ________		Assessor's title/position: ________	Assessor's signature: ________

Weight:	Mid upper arm circ.:	Fundal height:	Fetal heart sound?	Ultrasound done (since visit 1)?
______ kg	__________ cm	_______cm NA	Yes No NA	Yes (complete "tests") No

Since visit 1, have you smoked?	Since visit 1, have you drunk alcohol?	Since visit 1, have you used illegal drugs?
No Yes (cigarettes/day):	No Yes (drinks/week):	No Yes (specify):

<u>Since your last visit</u> ...

(*For questions below, write any conditions, treatments, tests in the "medical history", "treatments", "test results" section opposite)*

Have you had malaria?	Yes	No	NK
Have you taken any treatments to prevent malaria?	Yes	No	NK
Have you had any fever other than malaria?	Yes	No	NK
Have you had any vaccines?	Yes	No	NK
Have you had any blood transfusions?	Yes	No	NK
Have you had any other injections?	Yes	No	NK
Have you had any other condition (apart from malaria and fever)?	Yes	No	NK
Have you taken any traditional or herbal medicines?	Yes	No	NK
Have you taken any other treatments (including routine treatments - e.g. folic acid, iron supplements, multivitamins, deworming tablets)?	Yes	No	NK
Have you had any tests at a clinic (apart from an ultrasound)?	Yes	No	NK

For midwife to complete:

(For questions below, write any treatments and tests prescribed in "medical history" and "test results" section opposite)

At the clinic today, will this patient be prescribed one or more treatments?	Yes	No
At the clinic today, will this patient be given or prescribed one or more tests?	Yes	No

Visit 3			
Visit date: ________ dd mmm yy	Assessor's name: ________	Assessor's title/position: ________	Assessor's signature: ________

Weight:	Mid upper arm circ.:	Fundal height:	Fetal heart sound?	Ultrasound done (since visit 2)?
______ kg	____________ cm	________ cm NA	Yes No NA	Yes (complete "tests") No

Since visit 2, have you smoked?	Since visit 2, have you drunk alcohol?	Since visit 2, have you used illegal drugs?
No Yes (cigarettes/day):	No Yes (drinks/week):	No Yes (specify):

<u>Since your last visit</u> ...

(*For questions below, write any conditions, treatments, tests in the "medical history", "treatments", "test results" section opposite)*

Have you had malaria?	Yes	No	NK
Have you taken any treatments to prevent malaria?	Yes	No	NK
Have you had any fever other than malaria?	Yes	No	NK
Have you had any vaccines?	Yes	No	NK
Have you had any blood transfusions?	Yes	No	NK
Have you had any other injections?	Yes	No	NK
Have you had any other condition (apart from malaria and fever)?	Yes	No	NK
Have you taken any traditional or herbal medicines?	Yes	No	NK
Have you taken any other treatments (including routine treatments - e.g. folic acid, iron supplements, multivitamins, deworming tablets)?	Yes	No	NK
Have you had any tests at a clinic (apart from an ultrasound)?	Yes	No	NK

For midwife to complete:

(For questions below, write any treatments and tests prescribed in "medical history", "treatments" and "test results" section opposite)

At the clinic today, will this patient be prescribed one or more treatments?	Yes	No
At the clinic today, will this patient be given or prescribed one or more tests?	Yes	No

Visit 4			Registry ID:______________	Clinic ID: ______________
Visit date: ________ dd mmm yy	Assessor's name: ____________________		Assessor's title/position: ________	Assessor's signature: ____________
Weight: ______ kg	Mid upper arm circ.: _____________ cm	Fundal height: _______ cm NA	Fetal heart sound? Yes No NA	Ultrasound done (since visit 3)? Yes (complete "tests") No
Since visit 3, have you smoked? No Yes (cigarettes/day):		Since visit 3, have you drunk alcohol? No Yes (drinks/week):		Since visit 3, have you used illegal drugs? No Yes (specify):

Since your last visit ...
(For questions below, write any conditions, treatments, tests in the "medical history", "treatments", "test results" section opposite)

Have you had malaria?	Yes	No	NK
Have you taken any treatments to prevent malaria?	Yes	No	NK
Have you had any fever other than malaria?	Yes	No	NK
Have you had any vaccines?	Yes	No	NK
Have you had any blood transfusions?	Yes	No	NK
Have you had any other injections?	Yes	No	NK
Have you had any other condition (apart from malaria and fever)?	Yes	No	NK
Have you taken any traditional or herbal medicines?	Yes	No	NK
Have you taken any other treatments (including routine treatments)?	Yes	No	NK
Have you had any tests at a clinic (apart from an ultrasound)?	Yes	No	NK

For midwife to complete:
(For questions below, write any treatments and tests prescribed in "medical history" and "test results" section opposite)

At the clinic today, will this patient be prescribed one or more treatments?	Yes	No
At the clinic today, will this patient be given or prescribed one or more tests?	Yes	No

Visit 5				
Visit date: ________ dd mmm yy	Assessor's name: ____________________		Assessor's title/position: ________	Assessor's signature: ____________
Weight: ______ kg	Mid upper arm circ.: ___________ cm	Fundal height: _______ cm NA	Fetal heart sound? Yes No NA	Ultrasound done (since visit 4)? Yes (complete "tests") No
Since visit 4, have you smoked? No Yes (cigarettes/day):		Since visit 4, have you drunk alcohol? No Yes (drinks/week):		Since visit 4, have you used illegal drugs? No Yes (specify):

Since your last visit ...
(For questions below, write any conditions, treatments, tests in the "medical history", "treatments", and "test results" section opposite)

Have you had malaria?	Yes	No	NK
Have you taken any treatments to prevent malaria?	Yes	No	NK
Have you had any fever other than malaria?	Yes	No	NK
Have you had any vaccines?	Yes	No	NK
Have you had any blood transfusions?	Yes	No	NK
Have you had any other injections?	Yes	No	NK
Have you had any other condition (apart from malaria and fever)?	Yes	No	NK
Have you taken any traditional or herbal medicines?	Yes	No	NK
Have you taken any other treatments (including routine treatments - e.g. folic acid, iron supplements, multivitamins, deworming tablets)?	Yes	No	NK
Have you had any tests at a clinic (apart from an ultrasound)?	Yes	No	NK

For midwife to complete:
(For questions below, write any treatments and tests prescribed in "medical history" and "test results" section opposite)

At the clinic today, will this patient be prescribed one or more treatments?	Yes	No
At the clinic today, will this patient be given or prescribed one or more tests?	Yes	No

Registry ID	Clinic ID

Medical history

Condition(complete all "treatments" in section below)	Start/ diagnosis date	Duration	How was condition diagnosed? (tick all that apply)					Visit reported
	dd mmm yy/ how long ago	days/months/ years/ongoing	Clinical	Smear/ Microscopy	Rapid test	Swab	Other(specify) or NK	

Treatments

Part I. Routine treatments (Circle all visits during which routine treatments were reported or prescribed)

**Name of treatment**	Folic Acid	Iron Supplement	Folic acid + Iron Supplement	IPTp SP	Tetanus Toxoid	Mutivitamin
**Date Started** **dd mmm yy/ how long ago**						
**Visits reported/ prescribed**	**1 2 3 4 5 6 7 8**	**1 2 3 4 5 6 7 8**	**1 2 3 4 5 6 7 8**	**1 2 3 4 5 6 7 8**	**1 2 3 4 5 6 7 8**	**1 2 3 4 5 6 7 8**

Part II. Other treatments

Name of treatment *(complete all corresponding conditions in "medical history section" above)*	Indication	Start	Duration	Route*	Source of information**	Visit reported
		dd mmm yy/ how long ago	once, ongoing, # of days/months/ years			

* oral, rectal, injection, nasal, topical, ocular, per vagina **patient report (pt report), facility record (specify), patient's medical diary, other (specify)

Test results

Name of test	Date dd mmm yy	Result/unit
Haemoglobin		
Urinalysis		
HIV		
Syphilis/VDRL		

Medical history, treatments & tests, continued	Registry ID	Clinic ID

Medical history

Condition(complete all "treatments" in section below)	Start/ diagnosis date	Duration	How was condition diagnosed? (tick all that apply)					Visit reported
	dd mmm yy/ how long ago	days/months/ years/ongoing	Clinical	Smear/ Microscopy	Rapid test	Swab	Other(specify) or NK	

Treatments

Name of treatment *(complete all corresponding conditions in "medical history section" above)*	Indication	Start	Duration	Route*	Source of information**	Visit reported
		dd mmm yy/ how long ago	days/months/ years/ongoing			

* oral, rectal, injection, nasal, topical, ocular, per vagina **patient report (pt report), other record (specify), diary, other (specify)

Test results

Name of test	Date dd mmm yy	Result/unit

Comments and serious adverse events

Registry ID: ___________________________	Clinic ID: ______________________________

Comments

Topic	Comment	Initial/date

Serious adverse events

Circle any that apply:	Maternal death	Miscarriage	Stillbirth	Congenital anomaly	Neonatal death	Other
Date of SAE:<br><br>Information:						
Circle any that apply:	Maternal death	Miscarriage	Stillbirth	Congenital anomaly	Neonatal death	Other
Date of SAE:<br><br>Information:						

Completion guidelines for health-care workers

Registry ID	This is your 2 digit country code followed by the 2 digit site code followed by the sequential number at your clinic (out of a possible 9999 patients). Please remember the leading zeros/0's<br><br>e.g. Dodowa Health Research Centre, Ghana, patient number 12 will be: GHDO0012
Circling answers	Please indicate the answer to each multiple choice question by circling the correct information.
Date formats	The standard date format is dd mmm yy (e.g. 12th February 2009 is 12 FEB 09)<br><br>If you or the patient does not know part of the date you may put a line in its place. (e.g. -- / ---- / 09)<br><br>If a date is estimated/guessed then please use ± before the date. (e.g. ± 12 FEB 09)<br><br>If the you or the patient has approximate knowledge of the timescale only you may use x days ago, x weeks ago, x months ago, x years ago (e.g. 6 weeks ago)<br><br>Duration: leave the duration section blank until you know it. If a medical condition or treatment is ongoing at the time of delivery please write "ongoing' in the duration box.
Abbreviations	You may use well known abbreviations e.g. NK for not known, ND for not done, NA for not applicable. Otherwise write in full.
Questions to patients	Questions are to be asked of the patients as they are written. You will then follow the instructions and complete the relevant section.
Medical history, treatments	One line per item please unless an illness or treatment was stopped and then started again. Please indicate if illness/treatments were intermittent and then give the overall start and stop dates as above.
Information from other Sources	If you find any information from another source such as another clinic record form, diary, notes etc. please complete the registry as fully as possible.
Run out of space?	If you run out of space there is an extra page for medical history, treatments and tests.
Serious Adverse Events (SAEs)	In the event of an SAE (e.g. death, hospitalization, permanent damage or disability of mother or neonate, miscarriage, stillbirth, congenital anomaly, life-threatening events, including neonatal resuscitation) please complete the section on 'Extra Page & Comments'. Also, please alert the PI of the SAE and send any accompanying photographs within 24 hours.
Comments section	This is for additional relevant information or if you really don't know where to record something. Please use it sparingly.
Feedback	We would value any comments from you on these forms. Please give feedback to your registry contact person in writing or in person but not on the patient registry cards.

Thank you for your help with this important project and good luck!
