# Supplementary material for: Protocol for a drugs exposure pregnancy registry for implementation in resource-limited settings
Source: BMC Pregnancy Childbirth. 2012 Sep 3;12:89. doi: 10.1186/1471-2393-12-89 (PMC3500715; doi:10.1186/1471-2393-12-89)
Supplement: Additional file 2 — Case Record Form 2. Pregnancy Outcome Sheet Data capture form used during labor/delivery period or during assessment of mother and infant within 12 weeks after birth. [file 1471-2393-12-89-S2.pdf]

**Pregnancy registry**	**Pregnancy outcome data-sheet**	Mother's Registry ID:	Mother's initials:
		Mother's Clinic ID:	Mother's age (years):
		Baby's Registry ID:	Baby's Clinic ID:

Assessor's name: ____________	Assessor's position/title: ____________	Assessor's signature: ____________

Mother's medical history, treatments and test results (please circle one)
Already enrolled: since last ANC visit / Surface exam group: during pregnancy

How have you been and what treatments did you take even if unrelated to your pregnancy? Consider anything a health worker, traditional healer, birth attendant, shop-keeper, relative or friend has given/sold to you.

Have you had malaria?	Yes	No	NK
Have you had fever other than malaria?	Yes	No	NK
Have you taken any treatments to prevent malaria?	Yes	No	NK
Have you had any condition apart from malaria or fever?	Yes	No	NK
Have you had any vaccines?	Yes	No	NK
Have you taken any traditional or herbal medicines?	Yes	No	NK
Have you taken any routine treatments (e.g. folic acid, iron supplements, deworming tablets)?	Yes	No	NK
Have you taken any other treatment, apart from those mentioned above?	Yes	No	NK
Have you had any tests, including obstetric ultrasound, at a clinic?	Yes	No	NK

Medical history, treatments, and tests							
Condition (complete all "treatments" in section below)	Start /diagnosis date	Duration	How was condition diagnosed? (tick all that apply)				
	dd mmm yy/how long ago	ongoing, # of days/months/years	Clinical	Smear/ Microscopy	Rapid test	Swab	Other (specify) or NK

Name of treatment	Indication	Start date<br>dd mmm yy/how long ago	Duration<br>once, ongoing, # of days/months/years	Route*	Source of information**

* oral, rectal, injection, nasal, topical, ocular, per vagina **patient report (pt report), other record (specify), diary, other (specify)

Name of test: ____________		Name of test: ____________	
Date/how long ago	Result/unit	Date/how long ago	Result/unit
________ dd mmm yy		________ dd mmm yy	

Outcome of Current Pregnancy (use separate sheets for each baby if a multiple birth)		
Date of outcome: ________ dd mmm yy	Time of outcome ________ hh : mm	Place of outcome: ________
Date of assessment: ________ dd mmm yy	Time of assessment ________ hh : mm	Place of assessment: ________
Number of gestations: singleton or multiple, number: ____	Gestational age at birth outcome (weeks): ________	
Method for estimation of gestation age: LMP Ultrasound Other: ____________		
Type of delivery: Normal vaginal Forceps Vacuum Elective C-section Emergency C-Section Breech Elective/ Medical abortion		

Baby's Registry ID: ____________________						
Who delivered the baby?	Doctor	TBA	Nurse/midwife	Relative	Other (specify):________________	
What was the type of labour?	Spontaneous	Induced	Augmented	Elective C-section (no labour)	NK	
What was the birth outcome?	Infant alive	Infant dead, date of death: ____________________ dd mmm yy			NK	
What was the sex of the baby?	Male	Female	Ambiguous genitalia	NK		
If infant is dead: was the baby reported to be moving in the day prior to labour?	Yes	No	NK	NA		
If infant is dead: was a foetal heart sound detected at assessment before delivery?	Yes	No	NK	NA		
Were there complications at delivery (mother/child)?	No	Yes (describe): ____				

Assessment of the newborn/stillborn

Weight: ____________ g	Supine length: ____________cm	Head circumference: __________cm
Heart rate: _________beats/min or NA (if infant is dead)	Respiratory rate: ________ breaths/min or NA (if infant is dead)	
Has infant passed urine? Yes No NK NA (if infant is dead)	Has infant passed a stool? Yes No NK NA (if infant is dead)	

Examinations/examples of what to look for	Normal?		Description (remember to take photos of abnormalities)
Head and neck (including skull, fontanelles, eyes, ears, nose, jaw)	Yes	No	
Mouth, lips and palate (?thin/cleft)	Yes	No	
Chest (?shape, respiratory movements)	Yes	No	
Abdomen and anus (?masses/closure defect)	Yes	No	
Arms and legs (?length, shape, parts missing)	Yes	No	
Fingers and toes (including nails ?number, dangling, fused, shape/parts missing, abnormally large/small)	Yes	No	
Spine (?lumps or "cysts" or bulging in the back including the neck; thorax; lumbar area)	Yes	No	
Hips and genitalia (including urethra, testes, penile shaft, vagina, labia)	Yes	No	
Skin (?pale, blue, birth marks or any large "very red areas")	Yes	No	
Other abnormality or unusual finding. If so, describe and take photos			
Additional notes/comments			

If doctor did not assess infant, did a doctor confirm defect(s)?	No (why not?) ____________________	Yes (Doctor's name/signature/date): ______________ ____	NA
Was the baby referred?	No (why not?) ____________________	Yes (who to/where to): ______________________ ____	NA

General notes on completing this form

Use 'X 'or '√' or underline/circle a field. Ask questions about health/treatments/tests as they are written on the data sheet. If a woman indicates any illness, treatment or test (since last ANC visit if enrolled in registry already, or throughout pregnancy if surface exam group only) give details in the medical history/treatment/test tables below.

The standard date format is dd mmm yy e.g. 12 FEB 09. If part of the date is unknown put a line. e.g. -- / ---- / 09. If a date is estimated use ± in front e.g. ± 12 FEB 09. If only an approximate knowledge of timescale is known use x days ago, x weeks ago etc. e.g. 6 months ago. Leave the duration section blank until you know it. If a medical condition or treatment is ongoing at the time of delivery please write "ongoing' in the duration box.

One line per item unless it stopped and then started again. If intermittent condition/treatment, indicate this and write overall start/stop dates as above. Write in full apart from NK (not known), ND (not done), NA (not applicable).

If you find information from another source (e.g. another clinic record form, diary etc.) complete the form as fully as possible from this data. If updating the form with subsequent information, or correcting entries, neatly cross out the original and write the new. Initial/date any changes you make.

For multiple births use a separate form for each baby without duplicating mother's medical history/treatment/tests

Baby's Registry ID: ________________		
Second assessor's name: ____________	Second assessor's position/title: ____________	Second assessor's signature: ____________
Date of second assessment: ________ dd mmm yy	Time of second assessment: ________ hh : mm	Place of second assessment: ____________

Assessment of the newborn/stillborn

Weight: __________ g	Supine length: __________cm	Head circumference: __________cm
Heart rate: ________beats/min or NA (if infant is dead)	Respiratory rate: ________breaths/min or NA (if infant is dead)	
Has infant passed urine? Yes No NK NA (if infant is dead)	Has infant passed a stool? Yes No NK NA (if infant is dead)	

Examinations/examples of what to look for	Normal?		Description (remember to take photos of abnormalities)
Head and neck (including skull, fontanelles, eyes, ears, nose, jaw)	Yes	No	
Mouth, lips and palate (?thin/cleft)	Yes	No	
Chest (?shape, respiratory movements)	Yes	No	
Abdomen and anus (?masses/closure defect)	Yes	No	
Arms and legs (?length, shape, parts missing)	Yes	No	
Fingers and toes (including nails ?number, dangling, fused, shape/parts missing, abnormally large/small)	Yes	No	
Spine (?lumps or "cysts" or bulging in the back including the neck; thorax; lumbar area)	Yes	No	
Hips and genitalia (including urethra, testes, penile shaft, vagina, labia)	Yes	No	
Skin (?pale, blue, birth marks or any large "very red areas")	Yes	No	
Other abnormality or unusual finding. If so, describe and take photos			
Additional notes/comments			

General notes on completing this form

Use 'X 'or '√' or underline/circle a field. Ask questions about health/treatments/tests as they are written on the data sheet. If a woman indicates any illness, treatment or test (since last ANC visit if enrolled in registry already, or throughout pregnancy if surface exam group only) give details in the medical history/treatment/test tables below.

The standard date format is dd mmm yy e.g. 12 FEB 09. If part of the date is unknown put a line. e.g. -- / ---- / 09. If a date is estimated use ± in front e.g. ± 12 FEB 09. If only an approximate knowledge of timescale is known use x days ago, x weeks ago etc. e.g. 6 months ago. Leave the duration section blank until you know it. If a medical condition or treatment is ongoing at the time of delivery please write "ongoing' in the duration box.

One line per item unless it stopped and then started again. If intermittent condition/treatment, indicate this and write overall start/stop dates as above. Write in full apart from NK (not known), ND (not done), NA (not applicable).

If you find information from another source (e.g. another clinic record form, diary etc.) complete the form as fully as possible from this data. If updating the form with subsequent information, or correcting entries, neatly cross out the original and write the new. Initial/date any changes you make.

For multiple births use a separate form for each baby without duplicating mother's medical history/treatment/tests
