# Supplementary material for: Protocol for a drugs exposure pregnancy registry for implementation in resource-limited settings
Source: BMC Pregnancy Childbirth. 2012 Sep 3;12:89. doi: 10.1186/1471-2393-12-89 (PMC3500715; doi:10.1186/1471-2393-12-89)
Supplement: Additional file 3 — Case Record Form 3. Data capture form used during the confirmatory assessment of the infant for the suspected birth defect. To be completed by a relevant medical specialist (e.g. neonatologist, teratologist). [file 1471-2393-12-89-S3.pdf]

Confirmatory Exam for Congenital Abnormality by Specialist

If more than one baby fill a separate sheet for each baby (A, B, C for each child born - in order of birth)

Mother's Hospital ID: __________ **Mother's Registry ID Code:** __________

Mother's Initials: __________ **Mother's DOB/Age:** __________

Baby's Hospital ID: __________ **Baby's Registry ID Code:** __________

Place of Delivery: __________ **Place of Initial Assessment:** __________

DOB of infant: __________ **Date of Examination:** __________

Assessment of the new born (or stillborn baby) (Take photo if abnormalities detected)

Weight (g) __________ **Supine length (cm)** __________ **Head Circumference (cm)** __________

Heart rate (per minute): __________ **Respiratory rate (per minute):** __________

Reason for Referral of Child for Examination:

Pertinent History:

Examination Findings:	If Abnormal, Please describe:

Summary of examination findings:

Recommendations:

Clinician's Details:

Name of examining doctor: __________ **Qualification:** __________

Are you aware of the medicines taken by the mother during pregnancy?

Yes	No	Not Sure

Facility Name: __________

Telephone: __________ **Email:** __________ **Fax:** __________

Signature __________ **Date of report:** __________

Photograph of congenital anomaly provided? | Yes | No |

* Attach photograph to form -record ID code and clinic name with the photo
