# Supplementary material for: Protocol for a drugs exposure pregnancy registry for implementation in resource-limited settings
Source: BMC Pregnancy Childbirth. 2012 Sep 3;12:89. doi: 10.1186/1471-2393-12-89 (PMC3500715; doi:10.1186/1471-2393-12-89)
Supplement: Additional file 4 — Guidance on the use of medicines during pregnancy. A note given to recruited women on the prudent use of medicines during the course of the pregnancy. [file 1471-2393-12-89-S4.pdf]

Guidance on Taking Medicines During Pregnancy

Our common goal is for you to have a satisfying pregnancy period, safe delivery and a healthy baby. Understanding the following information about taking medicines in pregnancy can help.

- Some medicines are important to take while you are pregnant, but other medicines may not be safe.
- Always speak with the midwife, doctor or pharmacist if you are pregnant and starting a new medicine. Tell them that you are pregnant.
- Ask about the safety of the medicine in pregnancy. Discuss the need for taking a particular medicine in pregnancy with your doctor, midwife, or pharmacist.
- If you are pregnant, or wish to become pregnant, it is important that you take your folic acid tablets daily; It will help you and your baby during the pregnancy.
- Some over-the-counter medicines and medicines from vendors can be harmful to your baby. Ask the person who is selling you the medicines whether it is safe for you and the baby. If they do not know or are unsure, speak with a qualified midwife, doctor or pharmacist before taking the medicine.
- Traditional and herbal remedies are not always safe. Ask your midwife, doctor or pharmacist for advice about taking these while pregnant.
- If you experience any unusual effects (feel sick or bad) after taking your medicine, inform your midwife, doctor or pharmacist immediately.
- Everybody is different - one woman may take a harmful medicine and have a healthy baby and another woman may take a so-called safe medicine and end up having a child with a birth defect.
- Birth defects can occur naturally, even if you do not take any medicines. Sometimes they occur because of a family history or not enough of an important nutrient/vitamin.
